# Prolonged Jaundice in a Premature Breastfed Infant With Gilbert’s Syndrome

**DOI:** 10.1177/08903344241227226

**Published:** 2024-02-09

**Authors:** Frances Strobl, Melissa Ann Theurich

**Affiliations:** 1 School of Life Sciences, Technical University of Munich, Munich, Germany; 2 Chair of Public Health and Health Services Research, Institute for Medical Information Processing, Biometry and Epidemiology, Pettenkoffer School of Public Health, Faculty of Medicine, Ludwig-Maximilians-Universität München (LMU Munich), Munich, Germany

**Keywords:** breastfeeding, breastfeeding assessment, Gilbert’s syndrome, human milk, jaundice, Meulengracht syndrome, prolonged jaundice

## Abstract

**Introduction:**

Neonatal jaundice and prematurity pose significant barriers to breastfeeding in the first days of life. There is limited literature exploring the relationship between prolonged jaundice in breastfed infants and Gilbert’s (Meulengraght) syndrome. This case study describes the diagnostic and therapeutic challenges associated with Gilbert’s syndrome in a late preterm breastfed infant born in Germany.

**Main Issue:**

In this case report, an infant born to a primipara woman presented at 3 weeks postpartum to an International Board Certified Lactation Consultant. The initial assessment revealed a late preterm infant with inadequate weight gain and jaundice. The dyad received breastfeeding support and eventually achieved adequate weight gain; however, the infant’s jaundice persisted.

**Management:**

The consulting midwife suggested that the persistent jaundice was “breastmilk jaundice” and recommended temporarily interrupting breastfeeding. Due to a suspected family history of Gilbert’s Syndrome, the dyad was referred, instead, to a pediatric gastroenterologist. Pathologic liver disease was excluded, and genetic testing confirmed Gilbert’s Syndrome. At 6 months of age, the dyad was successfully breastfeeding and beginning complementary feeding.

**Conclusion:**

Genetic testing for Gilbert’s Syndrome should be considered for infants with prolonged jaundice and positive family history. Interruption or cessation of breastfeeding are not evidence-based recommendations, and current guidelines do not support these practices. Lactation professionals play a critical role in the management of breastfeeding for preterm infants with prolonged jaundice and should refer to specialists to rule out pathologic etiologies.

## Introduction

The relationship between prolonged jaundice in breastfed infants and Gilbert’s syndrome is presented in only a few earlier case studies and there is limited literature that explores the topic (Bancroft et al., 1998; Hong et al., 2010; Maruo et al., 2000; Monaghan et al., 1999; Saki et al., 2011). This case study describes the diagnostic and therapeutic challenges associated with Gilbert’s syndrome in a late premature breastfed infant living in Germany. It highlights the importance of lactation professionals in the care of infants with prolonged jaundice, including referral to specialists for exclusion of pathologic etiologies.

In Germany, 86% of infants are exclusively breastfed at birth; however, this number drops to 60% by 2 months of life (Theurich et al., 2019). One important barrier to breastfeeding within the first days of life is neonatal jaundice (Chiu et al., 2021). A significant proportion of infants develop jaundice, including 60% of full-term and 80% of preterm neonates, and jaundice is a common reason for hospital readmission (Olusanya et al., 2018). One of the most important clinical risk factors for hyperbilirubinemia and neurotoxicity in newborns is the degree of prematurity. For each week of gestation less than 40 weeks, the odds of developing hyperbilirubinemia increase by a factor of 1.7 (Flaherman et al., 2017). In 45% of the cases of neonatal jaundice, the cause of hyperbilirubinemia is unknown (Saki et al., 2011).

Neonatal jaundice occurs largely as a result of the breakdown of red blood cells (hemolysis). Bilirubin is transported in the blood as “unconjugated” bilirubin until the liver converts it into a conjugated form, which is then excreted into the gut. Very high levels of unconjugated bilirubin are toxic; infants with rapidly rising bilirubin levels are at heightened risk of brain damage (kernicterus). Therefore, any infant with high or rapidly rising serum bilirubin requires treatment. Infants with persistent jaundice should have their conjugated bilirubin level measured within the first 3–4 weeks of life to rule out serious liver conditions like cholestasis and biliary atresia (Kemper et al., 2022). A complete blood count, including reticulocyte count, is also helpful in determining etiology. If hyperbilirubinemia extends beyond 2 months of age, a diagnosis of Gilbert’s Syndrome (GS) should be considered (Flaherman et al., 2017).

Gilbert’s Syndrome is the most common hereditary congenital disorder resulting in increased levels of unconjugated bilirubin in the bloodstream (Saki et al., 2011). GS is caused by a mutation in the UGT1A1 gene which results in decreased activity of the uridine diphosphate-glucuronosyltransferase (UGT) enzyme. The main role of UGT is to detoxify bilirubin by conjugating it with glucuronic acid (Hong et al., 2010). GS alone cannot cause severe indirect hyperbilirubinemia, but may have a summative effect on bilirubin levels when combined with other factors like Glucose 6 phosphate dehydrogenase (G6PD) deficiency (Saki et al., 2011). GS alone does not require a secondary care referral, is largely asymptomatic, and does not influence life expectancy (King & Armstrong, 2019).

The aim of this case study is to describe a late preterm infant in Germany presenting to an International Board Certified Lactation Consultant (IBCLC) with prolonged jaundice, who was subsequently diagnosed with GS. This case study highlights GS as one potential cause for prolonged jaundice in premature breastfed infants. The parents of the patient described in this case study gave written consent for publication, and they read and approved the case study as submitted. We use the most recent World Health Organization (WHO) definitions for the terms “breastfeeding,” “exclusively breastfeeding,” and “mixed milk feeding under six months” (WHO & United Nations Children’s Fund [UNICEF], 2021).

## History and Observational Assessment

A 34-year-old married, primipara woman living in a large metropolitan city in Germany presented to an IBCLC at 3 weeks postpartum. The participant had a medical history of polycystic ovary syndrome, hypothyroidism, Gilbert’s Syndrome, antepartum hemorrhage and spontaneous labor resulting in the vaginal birth of a late preterm (36 + 0) male infant. She reported taking Enoxaprin by injection, and 100 mg Levothyroxine. In addition, she was taking prenatal vitamins, and fenugreek capsules. Her hemoglobin at hospital discharge was 10.5 g/dl.

The infant weighed 2485 g at birth and was jaundiced, but did not receive phototherapy in hospital because his bilirubin levels did not exceed the threshold required for phototherapy. A lingual frenectomy was performed in the hospital between the 2nd and 3rd day of life. The infant was mixed milk fed including infant formula supplementation twice while in hospital (30 ml daily) and he continued to be supplemented with infant formula post discharge. Oral Vitamin D (1 × 500 IU daily) was administered. A midwife conducted regular follow-up home visits.

**Figure 1.**
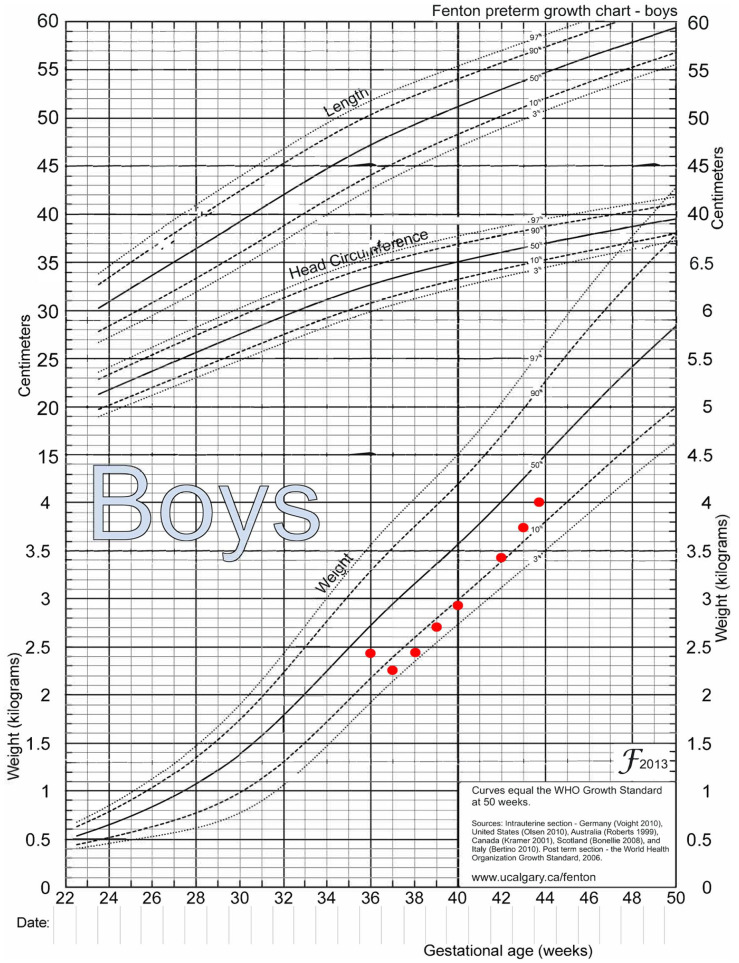
Fenton Growth Chart with Infant Weights by Age. *Note*. Adapted from Fenton and Kim (2013). Creative Commons License http://creativecommons.org/licenses/by/2.0.

The dyad presented at 3 weeks postpartum to the IBCLC due to difficulty with positioning and attachment. The initial assessment revealed a late preterm infant with limited buccal fat, ineffective transfer of milk at the breast, hyperextension of the neck, suspected unresolved ankyloglossia, umbilical hernia, inadequate weight gain and visible jaundice. At the initial visit, the infant’s weight had dropped from the 29^th^ percentile weight-for-age at birth to the 5th percentile, as shown on the Fenton preterm growth chart for boys (Figure 1).

## Management

The dyad was seen again 1 week later due to concerns of inadequate weight gain. At this point, the infant had gained some weight but remained below the minimum weight goal. An unresolved posterior tongue tie was suspected during the oral exam and breastfeeding observation. The dyad was referred to a pediatric dentist for assessment of suspected unresolved ankyloglossia. The parents were advised to continue breastfeeding on demand, and to supplement with expressed human milk as well as 120 ml formula (4 × 30 ml feedings daily) to improve weight gain. In addition, since the infant was late preterm, iron supplementation was recommended for prevention of iron deficiency anemia as per ABM protocols (Boies & Vaucher, 2016; Taylor, 2018). However, the infant did not receive iron supplementation due to advice from the pediatrician.

At 5 weeks postpartum, the infant was still jaundiced. A Coombs test for antibody-mediated hemolysis was negative. By 8 weeks of age, while weight gain had significantly improved, the infant continued to present with jaundice, including noticeably yellow skin and sclera. Therefore, small periodic supplements (1 × 10 ml infant formula, daily) were recommended following breastfeeding to help with bilirubin excretion, as per ABM protocol (Flaherman et al., 2017). At this point, the participant mentioned a positive history of GS in herself (she previously had undergone plasma bilirubin testing following fasting for diagnosis of GS). In addition, there was a potential family history of GS in the infant’s maternal grandfather and uncle. Therefore, the dyad was referred to a pediatric gastroenterologist for prolonged jaundice and suspected GS. A case summary and timeline can be found in Table 1.

**Table 1.** Timeline of Measured Bilirubin levels, Infant Weight, and Course of Events.

DOL	Bilirubin (mg/dl)	Weight (g)	Events
0		2485	Spontaneous vaginal delivery at 36+0 weeks, length: 46cm, APGAR 9/9/10, Coombs test negative, spontaneous labor, no phototherapy indicated, supplementation with infant formula
2-3			Lingual frenectomy in hospital
4			Discharge from hospital
6	11.6	2280	Supplementation with infant formula (30ml/day)
8	10.4		Initial visit with pediatrician
22		2700	Initial visit with IBCLC: inadequate weight gain as per Fenton preterm growth chart for boys, ongoing jaundice, stomach pain and gas. Stopped formula supplementation.
28		2930	Fed one bottle of infant formula (120ml) to improve weight gain.
33			Visit with pediatrician
37			Midwife notes increasing visible jaundice, recommends visit to pediatrician or hospital if infant becomes lethargic or refuses to breastfeed
40		3400	Infant weighed at home
42			Visit with pediatrician, blood test for bilirubin and ferritin
44	11.4, D Bil: 0.58	3580	Results from pediatrician
49		3740	
54		4000	Third visit with IBCLC, prolonged jaundice, mother expresses concerns about jaundice, says neighbors comment on the yellow color of the infant’s skin. IBCLC recommends pediatric gastroenterologist consult and visit with pediatrician for iron supplementation, bilirubin check. Recommends feeding small volumes of infant formula (10 ml) following breastfeeding.
57			Osteopathic consultation
84	8.0	4850	Pediatric gastroenterologist consult
93			Second frenectomy at pediatric dentist
177	1.2	6550	Follow-up with pediatric gastroenterologist to check aspartate transaminase

*Note.* DOL = day of life; D Bil = direct bilirubin.

## Outcomes

At 3 months of age, the infant was assessed by a pediatric gastroenterologist. Genetic testing confirmed a diagnosis of GS (UGT1A1*28 Homozygous, Genotype (TA)7/(TA)7). His bilirubin remained elevated and his aspartate transaminase was slightly elevated. Abdominal ultrasound confirmed normal visualization of his liver and gallbladder, and there were no pathological findings in his abdomen or urinary tract. Follow-up labs (transaminase, bilirubin, and iron) were recommended in 3 months. Shortly thereafter, a pediatric dentist dissected the lingual and labial frenulum.

Visual signs of jaundice completely resolved by 4 months of age. At 6 months of age, the infant was healthy, breastfeeding without supplementation of infant formula, and beginning complementary feeding. Slight iron deficiency anemia (IDA) was discovered on follow-up blood work at 6 months of age. Iron supplements were commenced following diagnosis of IDA.

## Discussion

This case study describes a first-time mother of a late preterm infant with multiple complex issues requiring intensive management. At the initial visit at 3 weeks of age, the infant was assumed to have “inadequate intake jaundice” due to ineffective milk transfer at the breast and inadequate weight gain. With improved breastfeeding and weight gain, the infant still had visibly yellow skin and sclera. Since GS had been diagnosed in the mother and amongst maternal family members, genetic screening for GS was considered. Consequently, the infant was referred to a specialist to determine the etiology of the prolonged jaundice. Following genetic testing at 12 weeks of age, the infant was diagnosed with GS.

The primary benefits of the diagnosis of GS in this case are: (1) the exclusion of pathologic etiologies for hyperbilirubinemia (Flaherman et al., 2017); (2) the continuation of breastfeeding without a need for infant formula supplementation; and (3) improved pharmacological management of potential future disease states, since hyperbilirubinemia in GS can interact with some medications.

Lactation consultants play an important role in the management of breastfeeding in jaundiced infants. In this case, infant jaundice did not fully resolve despite skilled support to improve breastfeeding and infant weight gain. The role of the IBCLC in this case was to protect sustained breastfeeding while liaising with the pediatric gastroenterologist, who could assess the etiology of prolonged jaundice. Lactation consultants are well positioned to facilitate screening for conjugated hyperbilirubinemia when they encounter persistently jaundiced neonates at 3 or 4 weeks of life.

Timely identification of pathologic conditions such as biliary atresia is crucial, since early identification and treatment improve outcomes. In clinical practice, prolonged jaundice is oftentimes attributed to “factors in human milk” without any work-up for hepatic or biliary causes. One strength of this case study is that the infant had a complete blood panel, liver function tests, several blood tests for bilirubin, abdominal ultrasound and genetic testing for GS, which together ruled out other potential causes for prolonged hyperbilirubinemia.

In 2010, Hong et al. published a case study on infants with GS in Korea who had severe neonatal hyperbilirubinemia that required treatment with phototherapy and phenobarbital (Hong et al., 2010). The authors noted that GS may be associated with more exaggerated hyperbilirubinemia if other risk factors—like prolonged fasting, surgery, or infection—are present. The association between GS and prolonged neonatal hyperbilirubinemia was first described in a cohort of neonates in the United States by Bancroft et al. (1998). Shortly afterwards, the same association was investigated in a cohort of 85 full-term infants in Scotland with unexplained hyperbilirubinemia (Monaghan et al., 1999). Monaghan et al. found that the incidence of familial hyperbilirubinemia genotypes (UGT1A1 7/7 and 5/7) were five times greater in very prolonged cases (ongoing elevated serum total bilirubin > 6 mg/dl at Day 28), compared to acute cases. They suggested that breastfed infants with the UGT1A1 7/7 genotype are more susceptible to hyperbilirubinemia and very prolonged jaundice compared to formula-fed infants (Monaghan et al., 1999).

Similarly, a case series of 17 breastfed Japanese infants by Maruo et al. (2000) suggested that GS may be an underlying cause of jaundice, and that components in human milk may trigger jaundice onset. The authors describe breastfeeding cessation as a contributing factor for normalization of bilirubin levels. Similar interpretation of findings were published in a more recent retrospective case-control study of 324 infants in China (Yang et al., 2022). However, a systematic review on human milk constituents and the development of “breast milk jaundice” states that “breast milk jaundice” is likely multifactorial, since no single constituent of human milk could yet explain all human milk jaundice cases observed (Gao et al., 2023). It is unclear, therefore, why these epidemiological studies interpret human milk as a causal risk factor for infant jaundice based solely on the risk differences between breastfed and formula fed groups. Indeed, UGT1A1 reaches normal adult activity by 3 months of age and jaundice typically resolves by this time, including in exclusively breastfeeding dyads. The case presented here exemplifies the resolution of hyperbilirubinemia with minimal formula supplementation and a gradual transition to exclusive breastfeeding, without need for breastfeeding cessation or a temporary interruption of breastfeeding.

There is limited literature on the influence of maternal GS with unmanaged hyperbilirubinemia on the breastfed infant, so we are unsure what potential influence the maternal diagnosis of GS would, in this case, have had on prolonged jaundice in this infant; however, this could be an interesting area for potential future research.

A secondary outcome in this case study was the development of IDA at 6 months of life. In late preterm breastfed infants, routine iron supplementation is recommended from birth by the WHO, American Academy of Pediatrics and others (Boies & Vaucher, 2016; Taylor, 2018).

In conclusion, taking family history regarding jaundice should be routine management in infants with prolonged jaundice. When a family history of GS is suspected, genetic screening for GS should be considered. Interruption and cessation of breastfeeding is associated with serious health risks in preterm infants. Current guidelines do not support interrupting or stopping breastfeeding in infants with prolonged jaundice (Kemper et al., 2022). Parents of infants with GS should be encouraged and supported to breastfeed.

## References

[bibr1-08903344241227226] Bancroft J. D. Kreamer B. Gourley G. R. (1998). Gilbert syndrome accelerates development of neonatal jaundice. The Journal of Pediatrics, 132(4), 656–660. 10.1089/bfm.2016.29031.egb 9580766

[bibr2-08903344241227226] Boies E. G. Vaucher Y. E. (2016). ABM Clinical Protocol #10: Breastfeeding the late preterm (34–36 6/7 weeks of gestation) and early term infants (37–38 6/7 weeks of gestation), second revision. Breastfeeding Medicine, 11(10), 494–500. 10.1089/bfm.2016.29031.egb 27830934

[bibr3-08903344241227226] Chiu Y. W. Cheng S. W. Yang C. Y. Weng Y. H. (2021). Breastfeeding in relation to neonatal jaundice in the first week after birth: Parents’ perceptions and clinical measurements. Breastfeeding Medicine, 16(4), 292–299. 10.1089/bfm.2020.0293 33666511

[bibr4-08903344241227226] Fenton T. R. Kim J. H. (2013). A systematic review and meta-analysis to revise the Fenton growth chart for preterm infants. BMC Pediatrics, 13, Article 59. 10.1186/1471-2431-13-59 PMC3637477 23601190

[bibr5-08903344241227226] Flaherman V. J. Maisels M. J. , & Academy of Breastfeeding Medicine. (2017). ABM Clinical Protocol #22: Guidelines for management of jaundice in the breastfeeding infant 35 weeks or more of gestation-revised 2017. Breastfeeding Medicine, 12(5), 250–257. 10.1089/bfm.2017.29042.vjf 29624434

[bibr6-08903344241227226] Gao C. Guo Y. Huang M. He J. Qiu X. (2023). Breast milk constituents and the development of breast milk jaundice in neonates: A systematic review. Nutrients, 15(10), Article 2261. 10.3390/nu15102261 PMC10224501 37242142

[bibr7-08903344241227226] Hong Y. S. Jin J. Y. Lee W. R. (2010). A case of Gilbert’s syndrome with severe neonatal hyperbilirubinemia. Journal of the Korean Society of Neonatology, 17(2), 266–269. 10.5385/jksn.2010.17.2.266

[bibr8-08903344241227226] Kemper A. R. Newman T. B. Slaughter J. L. Maisels M. J. Watchko J. F. Downs S. M. Grout R. W. Bundy D. G. Stark A. R. Bogen D. L. Holmes A. V. Feldman-Winter L. B. Bhutani V. K. Brown S. R. Maradiaga Panayotti G. M. Okechukwu K. Rappo P. D. Russell T. L. (2022). Clinical practice guideline revision: Management of hyperbilirubinemia in the newborn infant 35 or more weeks of gestation. Pediatrics, 150(3), Article e2022058859. 10.1542/peds.2022-058859 35927462

[bibr9-08903344241227226] King D. Armstrong M. J. (2019). Overview of Gilbert’s syndrome. Drug and Therapeutics Bulletin, 57(2), 27–31. 10.1136/dtb.2018.000028 30709860

[bibr10-08903344241227226] Maruo Y. Nishizawa K. Sato H. Sawa H. Shimada M. (2000). Prolonged unconjugated hyperbilirubinemia associated with breast milk and mutations of the bilirubin uridine diphosphate- glucuronosyltransferase gene. Pediatrics, 106(5), Article E59. 10.1542/peds.106.5.e59 11061796

[bibr11-08903344241227226] Monaghan G. McLellan A. McGeehan A. Li Volti S. Mollica F. Salemi I. Din Z. Cassidy A. Hume R. Burchell B. (1999). Gilbert’s syndrome is a contributory factor in prolonged unconjugated hyperbilirubinemia of the newborn. The Journal of Pediatrics, 134(4), 441–446. 10.1016/s0022-3476(99)70201-5 10190918

[bibr12-08903344241227226] Olusanya B. O. Kaplan M. Hansen T. W. R. (2018). Neonatal hyperbilirubinaemia: A global perspective. The Lancet. Child & Adolescent Health, 2(8), 610–620. 10.1016/S2352-4642(18)30139-1 30119720

[bibr13-08903344241227226] Saki F. Hemmati F. Haghighat M. (2011). Prevalence of Gilbert syndrome in parents of neonates with pathologic indirect hyperbilirubinemia. Annals of Saudi Medicine, 31(2), 140–144. 10.4103/0256-4947.77498 21403409 PMC3102472

[bibr14-08903344241227226] Taylor S. N. (2018). ABM Clinical Protocol #29: Iron, Zinc, and Vitamin D supplementation during breastfeeding. Breastfeeding Medicine, 13(6), 398–404. 10.1089/bfm.2018.29095.snt 30016173

[bibr15-08903344241227226] Theurich M. A. Davanzo R. Busck-Rasmussen M. Díaz-Gómez N. M. Brennan C. Kylberg E. Bærug A. McHugh L. Weikert C. Abraham K. Koletzko B. (2019). Breastfeeding rates and programs in Europe: A survey of 11 national breastfeeding committees and representatives. Journal of Pediatric Gastroenterology and Nutrition, 68(3), 400–407. 10.1097/MPG.0000000000002234 30562307

[bibr16-08903344241227226] World Health Organization & United Nations Children’s Fund. (2021). Indicators for assessing infant and young child feeding practices: Definitions and measurement methods. https://www.who.int/publications/i/item/9789240018389

[bibr17-08903344241227226] Yang Z. Lin F. Xu J. X. Yang H. Wu Y. H. Chen Z. K. Xie H. Huang B. Lin W. H. Wu J. P. Ma Y. B. Li J. D. Yang L. Y. (2022). UGT1A1*6 mutation associated with the occurrence and severity in infants with prolonged jaundice. Frontiers in Pediatrics, 10, https://doi.org/10.3389/fped.2022.1080212 10.3389/fped.2022.1080212 PMC9808393 36605758

